# A Japanese prospective multicenter study of urinary oxysterols in biliary atresia

**DOI:** 10.1038/s41598-021-84445-w

**Published:** 2021-03-02

**Authors:** Ken-ichiro Konishi, Tatsuki Mizuochi, Hajime Takei, Ryosuke Yasuda, Hirotaka Sakaguchi, Jun Ishihara, Yugo Takaki, Masahiro Kinoshita, Naoki Hashizume, Suguru Fukahori, Hiromichi Shoji, Go Miyano, Koichiro Yoshimaru, Toshiharu Matsuura, Yukihiro Sanada, Takahisa Tainaka, Hiroo Uchida, Yumiko Kubo, Hiromu Tanaka, Hideyuki Sasaki, Tsuyoshi Murai, Jun Fujishiro, Yushiro Yamashita, Masaki Nio, Hiroshi Nittono, Akihiko Kimura

**Affiliations:** 1 Department of Pediatrics and Child Health, Kurume University School of Medicine, 67 Asahi-machi, Kurume, 8300011 Japan; 2 Department of Pediatric Surgery, Graduate School of Medicine, The University of Tokyo, Tokyo, Japan; 3 Junshin Clinic Bile Acid Institute, Tokyo, Japan; 4 Department of Pediatric Surgery, Kurume University School of Medicine, Kurume, Japan; 5 Department of Pediatrics, Juntendo University School of Medicine, Tokyo, Japan; 6 Department of Pediatric General and Urogenital Surgery, Juntendo University School of Medicine, Tokyo, Japan; 7 Department of Pediatric Surgery, Graduate School of Medical Sciences, Kyushu University, Fukuoka, Japan; 8 Department of Surgery, Division of Gastroenterological, General and Transplant Surgery, Jichi Medical University, Shimotsuke, Japan; 9 Department of Pediatric Surgery, Nagoya University Graduate School of Medicine, Nagoya, Japan; 10 Department of Pediatric Surgery, Tohoku University Graduate School of Medicine, Sendai, Japan; 11 School of Pharmaceutical Sciences, Health Sciences University of Hokkaido, Hokkaido, Japan

**Keywords:** Mass spectrometry, Diagnostic markers, Bile duct disease, Liver diseases, Sterols

## Abstract

Diagnosis of biliary atresia (BA) can involve uncertainties. In the present prospective multicenter study, we considered whether urinary oxysterols represent a useful marker for diagnosis of BA in Japanese children. Subjects under 6 months old at 7 pediatric centers in Japan were prospectively enrolled, including patients with cholestasis and healthy controls (HC) without liver disease. Patients with cholestasis constituted 2 groups representing BA patients and others with cholestasis from other causes (non-BA). We quantitatively analyzed 7 oxysterols including 4β-, 20(S)-, 22(S)-, 22(R)-, 24(S)-, 25-, and 27-hydroxycholesterol by liquid chromatography/electrospray ionization-tandem mass spectrometry. Enrolled subjects included 14 with BA (median age 68 days; range 26–170) and 10 non-BA cholestatic controls (59; 14–162), as well as 10 HC (57; 25–120). Total urinary oxysterols were significantly greater in BA (median, 153.0 μmol/mol creatinine; range 24.1–486.7; *P* < 0.001) and non-BA (36.2; 5.8–411.3; *P* < 0.05) than in HC (2.7; 0.8–7.6). In patients with BA, urinary 27-hydroxycholesterol (3.61; 0.42–11.09; *P* < 0.01) was significantly greater than in non-BA (0.71; 0–5.62). In receiver operating characteristic (ROC) curve analysis for distinguishing BA from non-BA, the area under the ROC curve for urinary 27-hydroxycholesterol was 0.83. In conclusion, this first report of urinary oxysterol analysis in patients with BA indicated that 27-hydroxycholesterol may be a useful marker for distinguishing BA from other causes of neonatal cholestasis.

## Introduction

Biliary atresia (BA), considered the most life-threatening hepatobiliary disorder in children, results from inflammation with fibrosis of the intra- and extrahepatic biliary tree^1^. Without treatment BA progresses to liver cirrhosis leading to death within 2 years. Occurring in 1 of 8000 to 18,000 live births, BA appears to be more frequent in Asians and Africans than in Europeans, and more common in girls than boys^2^. BA is the most common cause of neonatal cholestasis, leading to 40–50% of liver transplants in children^3^. Idiopathic neonatal hepatitis, neonatal intrahepatic cholestasis caused by citrin deficiency (NICCD), and Alagille syndrome are among other causes of neonatal cholestasis that can confound the diagnosis of BA, even though characteristics of these diseases tend to differ. The only definitive diagnostic procedure in BA is surgical cholangiography^1^. Increased age at surgery, Kasai portoenterostomy, had a progressive and sustained deleterious effect on the results of the operation until adolescence. If performed within the first 45 days of life, Kasai portoenterostomy is associated with 65.5% survival with the native liver at 2 years of age^4^. Previous studies analyzing various metabolites or proteins, including serum hyaluronic acid, apolipoprotein C3, interleukin-6, interleukin-8, urine sulfate conjugated bile acid, and stool secondary bile acids have identified potential diagnostic or screening markers for BA^5–9^. However, lack of prospective validation in these studies limits applicability of the findings. Recently, serum matrix metalloproteinase-7 (MMP-7) has shown promise in diagnosing BA^10–13^. However, we know of no studies of serum MMP-7 in Japanese patients with BA.

Together with other important markers related with liver injury and cholestasis, bile acids have been investigated in screening for or discriminating among cholestatic liver disorders including BA^8,14^. However, which specific bile acids are important as potential diagnostic markers of BA is not clear. We focused on oxysterols, which are 27-carbon cholesterol derivatives that can result from either enzymatic reactions or oxidation by free radicals^15^. Oxysterols are important as bile acid precursors and also as readily transportable forms of cholesterol. An example is 27-hydroxycholesterol, which is important as a chenodeoxycholic acid precursor in the acidic pathway of bile acid synthesis^16^. Oxysterols have been suspected to participate in the pathogenesis of some diseases including liver disease^17–19^. Few reports have considered oxysterols in children. Meng et al. reported that severe cholestatic liver disease in children may accelerate output of 24(S)-hydroxycholesterol from the brain, which can be detected in serum and urine. 24(S)-Hydroxycholesterol therefore may be clinically useful to measure in children with cholestatic liver disease^20^. So far we know of no prior studies considering oxysterols as potentially useful markers for BA. Recently, we reported that urinary oxysterols follow a developmental pattern in healthy children; deviations might suggest pediatric liver disease^21^. We hypothesized that urinary oxysterol determinations could help in distinguishing BA from other causes of neonatal cholestasis such as NICCD and Alagille syndrome, and examined the utility of oxysterol measurements in this prospective multicenter Japanese study.

## Results

### Study population

We enrolled 24 patients including 14 with BA and 10 with cholestasis from other causes (non-BA), along with 10 healthy controls (HC). Non-BA diagnoses included NICCD in 3 patients, Alagille syndrome in 2, unknown in 2, neonatal leukemia in 1, Dubin-Johnson syndrome in 1, and hypothyroidism in 1. Two subjects with BA and 3 with non-BA were also enrolled in our previous study^21^, as were 3 HC. Characteristics of BA and non-BA patients are shown in Table 1. Serum GGT was significantly greater in BA than in non-BA, while gender, age, serum alanine aminotransferase, total and direct bilirubin, total bile acids, and total cholesterol showed no significant differences (Table 1). Ages among BA (median 68 days; range 26–170), non-BA (59; 14–162), and HC (57; 25–120) subjects showed no significant differences.

**Table 1:** Patient characteristics in BA and non-BA.

	BA Median (range)	Non-BA Median (range)	*P* value
Number of subjects	14	10	
Gender (male/female)	6/8	6/4	0.68
Age, days	68 (26–170)	59 (14–162)	0.98
Alanine aminotransferase, U/L	101 (17–238)	69 (26–208)	0.36
γ-Glutamyltranspeptidase, U/L	620 (329–1175)	158 (47–279)	< 0.001
Total bilirubin, mg/dL	7.8 (4.6–13.1)	6.8 (3.3–22.7)	0.60
Direct bilirubin, mg/dL	5.2 (2.7–8.9)	3.7 (1.7–16.5)	0.33
Total bile acids, μmol/L^a^	109 (54–142)	182 (52–343)	0.31
Total cholesterol, mg/dL^b^	194 (109–313)	173 (120–224)	0.58

*BA* biliary atresia, *non-BA* non-biliary atresia cholestatic controls.

^a^BA and non-BA include 10 and 7 samples, respectively.

^b^BA and non-BA include 12 and 8 samples, respectively.

### Qualitative and quantitative urinary oxysterol analysis

**Figure 1:**
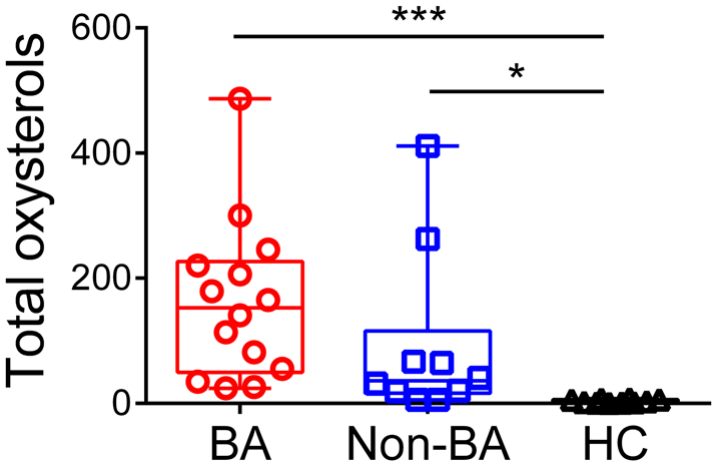
Urinary total oxysterols among BA, non-BA, and HC. Urinary total oxysterols are shown for subjects with biliary atresia (BA) and non-biliary atresia cholestatic controls (non-BA), as well as healthy controls (HC). Units are μmol/mol creatinine. Horizontal lines in the middle of boxes indicate medians, while tops and bottoms of boxes represent 75th and 25th percentiles, respectively. Whiskers above and below boxes represent maximum and minimum, respectively. *P < 0.05; ***P < 0.001.

First, we compared urinary total oxysterols among BA, non-BA, and HC subjects. Urinary total oxysterols were significantly greater in BA (median 153.0 μmol/mol creatinine; range 24.1–486.7; *P* < 0.001) and non-BA (36.2; 5.8–411.3; *P* < 0.05) than in HC (2.7; 0.8–7.6), while no significant difference was evident between BA and non-BA (*P* = 0.42; Fig. 1).

**Figure 2:**
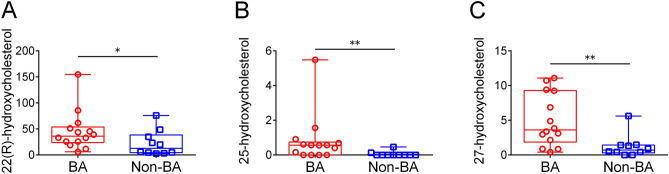
Urinary 22(R)-hydroxycholesterol, 25-hydroxycholesterol, and 27-hydroxycholesterol: BA vs. non-BA. Urinary 22(R)-hydroxycholesterol (**A**), 25-hydroxycholesterol (**B**), and 27-hydroxycholesterol (**C**) are shown for biliary atresia (BA) and non-biliary atresia cholestatic controls (non-BA). Units are μmol/mol creatinine. Horizontal lines in the middle of boxes indicate medians, while tops and bottoms of boxes represent 75th and 25th percentiles, respectively. Whiskers above and below boxes represent maximum and minimum, respectively. *P < 0.05; **P < 0.01.

Next, we compared 7 urinary oxysterols between BA and non-BA. In patients with BA, 22(R)-hydroxycholesterol (median 36.22 μmol/mol creatinine; range, 6.12–155.0; *P* < 0.05), 25-hydroxycholesterol (0.56; 0–5.49; *P* < 0.01) and 27-hydroxycholesterol (3.61; 0.42–11.09; *P* < 0.01) were significantly greater than in non-BA (12.91; 2.7–75.87, 0; 0–0.46, and 0.71; 0–5.62, respectivel*y*; Fig. 2A–C). Urinary 4β-hydroxycholesterol and 24(S)-hydroxycholesterol showed no significant differences between the 2 groups (Supplementary Fig. 1A,B), while 20(S)-hydroxycholesterol and 22(S)-hydroxycholesterol were not detected in either group.

### ROC analysis

**Figure 3:**
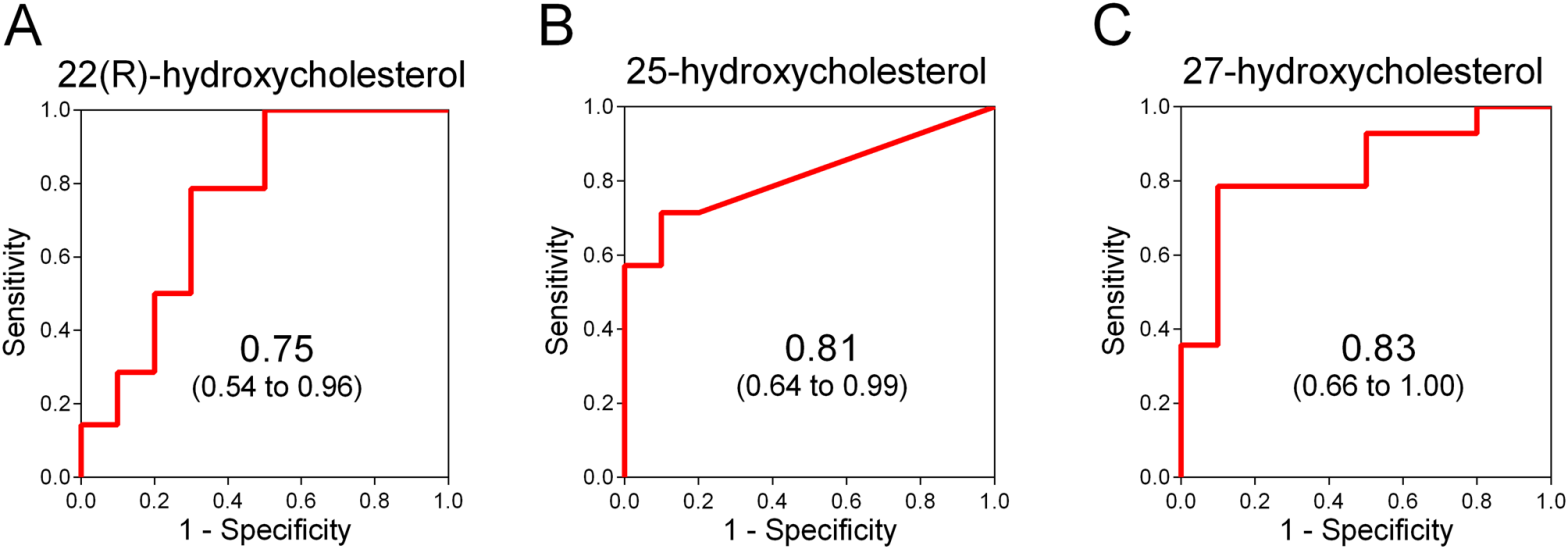
ROC curves for urinary 22(R)-hydroxycholesterol, 25-hydroxycholesterol, and 27-hydroxycholesterol. Receiver operating characteristic (ROC) curves for urinary 22(R)-hydroxycholesterol (**A**), 25-hydroxycholesterol (**B**) and 27-hydroxycholesterol (**C**) were obtained by plotting sensitivity against 1-specificity. Sensitivity and specificity were calculated using the results from 14 patients with biliary atresia and 10 with non-biliary atresia cholestatic controls. Areas under ROC curves are shown with 95% confidence intervals.

To assess diagnostic accuracy for BA, we performed receiver operating characteristic (ROC) analysis for urinary 22(R)-hydroxycholesterol, 25-hydroxycholesterol, and 27-hydroxycholesterol. In ROC analysis for distinguishing BA from non-BA, areas under ROC curves (AUC) for urinary 22(R)-hydroxycholesterol, 25-hydroxycholesterol, and 27-hydroxycholesterol were 0.75 (95% confidence interval 0.54–0.96), 0.81 (0.64–0.99), and 0.83 (0.66–1.00), respectively (Fig. 3A–C).

### Diagnostic accuracy of urinary 27-hydroxycholesterol in BA

In the ROC analysis for distinguishing BA from non-BA patients, an optimal cut‐off value of 2.17 μmol/mol creatinine of urinary 27-hydroxycholesterol demonstrated sensitivity, specificity, and positive and negative predictive values of 79%, 90%, and 92% and 75%, respectively.

### Analysis of factors affecting urinary 27-hydroxycholesterol in patients with BA

**Figure 4:**
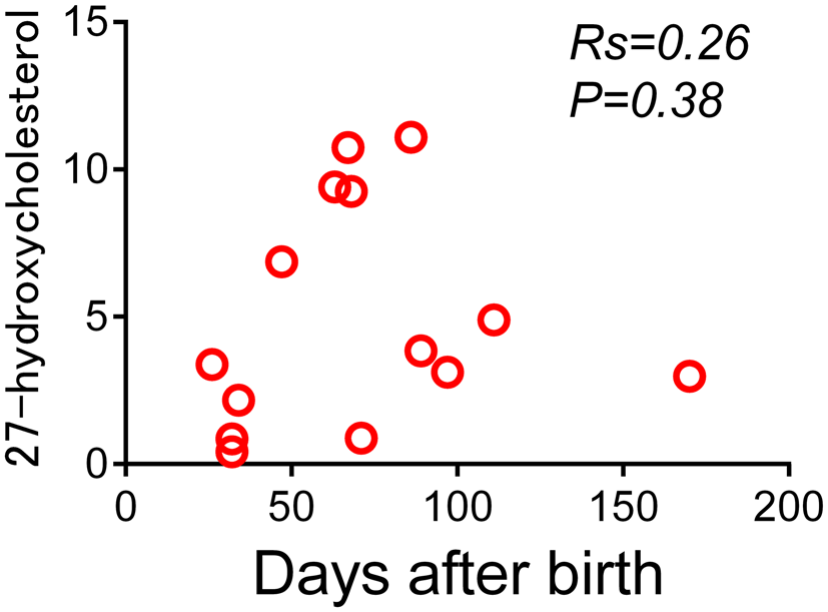
Correlation between urinary 27-hydroxycholesterol and age. Correlation is shown between urinary 27-hydroxycholesterol and age (days after birth) in patients with biliary atresia. *Rs*, Spearman's rank correlation coefficient.

Seeking factors that might affect urinary 27-hydroxycholesterol in patients with BA, we performed Spearman’s rank correlation test concerning urinary 27-hydroxycholesterol for patient age and various blood test results. No correlation with 27-hydroxycholesterol was found in BA patients for age (days after birth), serum alanine aminotransferase, γ-glutamyltransferase (GGT), total or direct bilirubin, total bile acids, or total cholesterol (Fig. 4; Supplementary Fig. 2A–F).

### Urinary bile acid analysis concerning the acidic pathway in BA and non-BA cholestasis

In patients with BA, 3β-hydroxy-5-cholenoic acids (Δ^5^-3β-ols; median, 6.29 mmol/mol creatinine; range, 1.01–7.82; *P* = 0.09) and 3β,7α-dihydroxy-5-cholenoic acids (Δ^5^-3β,7α-diols; 0.75; 0.12–1.10; *P* = 0.06) were greater than in non-BA (4.51; 0.40–6.33 and 0.51; 0.06–1.26, respectively; Supplementary Fig. 3A,B).

## Discussion

Our prospective multicenter study of Japanese infants indicated that urinary 27-hydroxycholesterol may be a useful marker for distinguishing BA from other causes of neonatal cholestasis.

Many previous studies concerning bile acids have suggested potential diagnostic or screening markers for BA. Being nonspecific, however, these markers have been unable to distinguish BA from other cholestasis. As an alternative we focused on oxysterols, which are bile acid precursors. We examined urinary oxysterols in BA, non-BA, and HC subjects in order to assess oxysterols as diagnostic markers for BA. Urinary 22(R)-hydroxycholesterol, 25-hydroxycholesterol, and 27-hydroxycholesterol in BA were significantly greater than in non-BA. Urinary 22(R)-hydroxycholesterol and 27-hydroxycholesterol were detected in all BA patients, while 25-hydroxycholesterol was not detected in some. The relative paucity of 25-hydroxycholesterol in humans^22^ makes urinary 25-hydroxycholesterol harder to detect than 27-hydroxycholesterol. Further, ROC analysis assigned urinary 27-hydroxycholesterol a more favorable AUC value than 22(R)-hydroxycholesterol and 25-hydroxycholesterol. Urinary 27-hydroxycholesterol thus is likely to be a better diagnostic marker for BA than 22(R)-hydroxycholesterol or 25-hydroxycholesterol.

27-Hydroxycholesterol is produced upon hydroxylation of cholesterol by the enzyme sterol 27-hydroxylase (CYP27A1)^23^. Hepatic CYP27A1 catalyzes the first step of the acidic pathway of bile acid synthesis as well as an intermediate step in the classical pathway^16^. The acidic pathway originates in the vascular endothelium, fibroblasts, and macrophages, with side chain 27-hydroxylation of cholesterol catalyzed by the mitochondrial sterol CYP27A1^24^. Mechanisms regulating CYP27A1 are unclear; a feedback mechanism similar to that for sterol 7-hydroxylase has not been detected in humans^25^. Measuring rates of plasma appearance of infused deuterated oxysterols in patients with chronic liver disease, Crosignani et al. concluded that the classical pathway is impaired while the acidic one is preserved^24^. In particular, severe liver fibrosis is associated with classical pathway suppression and acidic pathway enhancement^26^. Increased production of 27-hydroxycholesterol, which reflects the latter pathway, is suggestive of fibrosis^26,27^. In children with BA, fibrosis progresses more rapidly than other cholestatic liver diseases^1,28^, so highly elevated urinary 27-hydroxycholesterol should be a particularly useful diagnostic marker for BA. We also suspect that 27-hydroxycholesterol may have adverse biologic effects, considering that they are cholesterol oxidation products excreted in the urine as unusual bile acids^29,30^.

Since 27-hydroxycholesterol is a precursor of bile acids in the acidic pathway^16^, we specifically evaluated bile acids synthesized only via that pathway, comparing the urinary Δ^5^-3β-ol and the Δ^5^-3β,7α-diol groups between patients with BA and those with non-BA. In patients with BA, Δ^5^-3β-ols and Δ^5^-3β,7α-diols were greater than in non-BA patients. This data supports the view that 27-hydroxycholesterol is increased in BA because of acidic pathway upregulation. Unfortunately, we could not evaluate oxysterols in the classical pathway such as 7α-hydroxycholesterol, 7β-hydroxycholesterol, and 7-oxo-cholesterol because of previously reported oxidation effects^21,31^. 7α-Hydroxy-4-choesten-3-one, which reflects the condition of classical pathway, also was not examined^32^. We did measure cholic acid and 3β,7α,12α-trihydroxy-5-cholenoic acid, which are biosynthesized only by the classical pathway; no significant differences were evident between patient groups (data not shown). We plan to develop a method for evaluating the classical pathway by measuring 7α-hydroxycholesterol, 7β-hydroxycholesterol, 7-oxo-cholesterol and 7α-hydroxy-4-choesten-3-one in similar patient groups in the future.

We also investigated possible associations in BA patients between urinary 27-hydroxycholesterol and various factors including age and blood test results. No correlation was found between 27-hydroxycholesterol and age and or blood test results. Yang et al. reported that MMP-7 correlates positively with age and GGT^10^. Since early diagnosis is important in BA, by 60 days of age if possible, urinary 27-hydroxycholesterol appears superior to MMP-7 in this respect because it is not influenced by age or GGT. However, in previous reports, MMP-7 showed more favorable AUC values in distinguishing BA from non-BA than urinary 27-hydroxycholesterol did in this study^10–13^. In this study, BA subjects under 60 days of age included only 5 patients. Proving that 27-hydroxycholesterol is a better marker than serum MMP-7 in early diagnosis of BA ultimately will require study of more infants less than 60 days old with BA. Combining urinary 27-hydroxycholesterol with other useful markers such as serum MMP-7 and GGT might be the best approach to early and accurate diagnosis of BA.

We favor urinary 27-hydroxycholesterol as a noninvasive marker for distinguishing BA from other causes of neonatal cholestasis, because urine samples are easy to obtain from children. Additionally, urinary 27-hydroxycholesterol might be predictive of prognosis and need for liver transplantation after Kasai portoenterostomy because urinary 27-hydroxycholesterol appears linked to severe liver fibrosis in BA. In the future, we would like to specifically evaluate oxysterols such as urinary 27-hydroxycholesterol as a marker of need for liver transplantation following Kasai portoenterostomy in BA.

Even though our present research offers the strength of a prospective multicenter study, a number of limitations are evident. First, relatively small numbers of BA and non-BA subjects were enrolled. In particular, only 5 BA patients under 60 days of age were enrolled. Second, our samples included only Japanese subjects, so our findings should not be generalized to other Asian countries or different ethnic groups. Similar investigations in various other patient populations and ethnic groups are needed.

## Conclusions

To our knowledge, this is the first report of oxysterol analysis in patients with BA. We found that urinary 27-hydroxycholesterol may contribute importantly to distinguish BA from other causes of neonatal cholestasis.

## Materials and methods

### Study design and ethical considerations

This prospective multicenter observational study was conducted within the framework of the Investigation of Oxysterol and Bile Acid Analyses for Pediatric Patients with Liver Disease and Healthy Children in Japan, which includes 7 collaborating Japanese pediatric centers caring for patients with BA, together with the Junshin Clinic Bile Acid Institute, which has expertise in and resources for oxysterol and bile acid analyses in human specimens. The study protocol complied with the ethical guidelines of the Declaration of Helsinki (2013 revision) and was approved by the Ethics Committee at Kurume University and all participating centers. Written informed consent was obtained from enrolled subjects’ parents.

### Study subjects, definitions, and criteria

Subjects prospectively enrolled between November 2016 and August 2019 included patients under 6 months old from the 7 pediatric centers who had cholestasis, as well as HC without liver disease. Patients with cholestasis were divided into groups representing BA and non-BA. Urine samples from all enrolled subjects were analyzed. BA was diagnosed from the presence of fibrotic obstruction of extrahepatic biliary remnants in tissues excised after surgical cholangiography. Non-BA cholestasis was defined by (1) cholestatic liver diseases diagnosed by clinical findings and/or genetic analysis (including cholestasis of unknown cause), plus (2) serum direct bilirubin exceeding 1.5 mg/dL at sample collection. Patient samples were collected at the time of clinical diagnosis of BA or an alternative disease, prior to surgical cholangiography, Kasai portoenterostomy, or biliary drainage. Patient characteristics and serum laboratory results in BA and non-BA groups were obtained from medical records and clinical interviews. Demographic, clinical, and laboratory features considered included age, gender, and routine blood test results. Samples were stored under – 20 °C until oxysterol analysis.

### Urinary oxysterol analysis by LC/ESI–MS/MS

We quantitatively analyzed 7 types of oxysterols including 4β-hydroxycholesterol (cholest-5-en-3β,4β-diol), 20(S)-hydroxycholesterol (cholest-5-en-3β,20S-diol), 22(S)-hydroxycholesterol (cholest-5-en-3β,22S-diol), 22(R)-hydroxycholesterol (cholest-5-en-3β,22R-diol), 24(S)-hydroxycholesterol (cholest-5-en-3β,24S-diol), 25-hydroxycholesterol (cholest-5-en-3β,25-diol), and 27-hydroxycholesterol (cholest-5-en-3β,26-diol) using liquid chromatography/electrospray ionization-tandem mass spectrometry (LC/ESI–MS/MS) according to a previously reported method^21,31^. Urinary concentrations of individual oxysterols were corrected for creatinine concentration and are expressed as micromoles per moles of creatinine^21,31^.

Our quantitative analysis of urinary bile acids by LC/ESI–MS/MS is described in the Supplementary Methods.

### Oxysterol sample preparation

Urine samples were prepared by enzymatic hydrolysis of oxysterol conjugates according to a reported method^20,21,33,34^. Forty microliters of reagent A and ten microliters of reagent B from the Glufatase set were added to 200 µL of sample. The mixture was vortex-agitated for 10 s and allowed to stand for 5 min. After centrifugation for 5 min at 1500 rpm, 200 µL of supernatant was transferred to a 2-mL glass test tube along with 1 pmol of d_6_-25-hydroxycholesterol. Twenty microliters of acetate buffer from the Glufatase set were added, followed by 5 µL of glufatase enzyme from the Glufatase set and 5 µL of sulfatase type H-2. Derivatization to the nicotinyl ester was performed according to a previously reported method^35^ with minor modifications. Briefly, sterols were added to 100 µL of derivitization reagent (80 mg of nicotinic acid, 30 mg of *N*,*N*-dimethyl-4-aminopyridine, and 100 mg of 1-[3-dimethylaminopropyl]-3-ethylcarbodiimide hydrochloride in 1 mL of *N*,*N*-dimethylformamide). Samples were heated at 60 °C for 1 h, followed by addition of 1 mL of distilled water and 1 mL of hexane. After vortex-agitation of the mixture for approximately 2 min, nicotinyl ester derivatives were obtained by centrifugation for 5 min at 1000 rpm. The hexane layer was subjected to evaporation and reconstituted with 200 µL of acetonitrile. Aliquots of 10 µL were injected into the LC/ESI–MS/MS system.

Chemicals and reagents, preparation of standard solutions, and the LC/ESI–MS/MS analysis required for this oxysterol analysis are detailed in our previous report^21^.

### Laboratory tests

In BA and non-BA groups, serum alanine aminotransferase, GGT, total and direct bilirubin, and total cholesterol were examined. Serum total bile acids were determined in these patients using the 3α-hydroxysteroid dehydrogenase enzymatic method.

### Urinary bile acid analysis by LC/ESI–MS/MS concerning the acidic pathway

We specifically evaluated urinary bile acids synthesized only via the acidic pathway, comparing urinary Δ^5^-3β-ols and Δ^5^-3β,7α-diols between patients with BA and those with non-BA cholestasis. Detailed methods of urinary bile acid analysis for detecting Δ^5^-3β-ols and Δ^5^-3β,7α-diols are given in the Supplementary Methods^36^.

### Statistical analysis

Continuous variables are given as the median followed by the minimum and the maximum, while categorical variables are stated as the number of subjects demonstrating the variable. Fisher’s exact test, the Mann–Whitney *U* test, the Kruskal–Wallis test, Dunn's multiple comparison test, or Spearman’s rank correlation test was applied where appropriate. Diagnostic accuracy of an assay was evaluated by ROC analysis. Statistical analyses were carried out with GraphPad Prism (version 6.05; GraphPad Software, San Diego, CA, USA), which also was used to produce related figures. Significance tests were two-sided, and a *P* value below 0.05 was accepted as evidence of statistical significance^21^.

## Supplementary Information

Supplementary Information.

## Data Availability

The data that support the findings of this study are available on request from the corresponding author.
