# Supplementary material for: A Japanese prospective multicenter study of urinary oxysterols in biliary atresia
Source: Sci Rep. 2021 Mar 2;11:4986. doi: 10.1038/s41598-021-84445-w (PMC7925559; doi:10.1038/s41598-021-84445-w)
Supplement: Supplementary file 1 — Supplementary Information. [file 41598_2021_84445_MOESM1_ESM.pdf]

A Japanese prospective multicenter study of urinary oxysterols in biliary atresia

Ken-ichiro Konishi,[1,2] Tatsuki Mizuochi,[1,*] Hajime Takei,[3] Ryosuke Yasuda,[1] Hirotaka Sakaguchi,[1] Jun Ishihara,[1] Yugo Takaki,[1] Masahiro Kinoshita,[1] Naoki Hashizume,[4] Suguru Fukahori,[4] Hiromichi Shoji,[5] Go Miyano,[6] Koichiro Yoshimaru,[7] Toshiharu Matsuura,[7] Yukihiro Sanada,[8] Takahisa Tainaka,[9] Hiroo Uchida,[9] Yumiko Kubo,[10] Hiromu Tanaka,[10] Hideyuki Sasaki,[10] Tsuyoshi Murai,[11] Jun Fujishiro,[2] Yushiro Yamashita,[1] Masaki Nio,[10] Hiroshi Nittono,[3] and Akihiko Kimura [1]

1. Department of Pediatrics and Child Health, Kurume University School of Medicine, Kurume, Japan
2. Department of Pediatric Surgery, Graduate School of Medicine, The University of Tokyo, Tokyo, Japan
3. Junshin Clinic Bile Acid Institute, Tokyo, Japan
4. Department of Pediatric Surgery, Kurume University School of Medicine, Kurume, Japan
5. Department of Pediatrics, Juntendo University School of Medicine, Tokyo, Japan
6. Department of Pediatric General and Urogenital Surgery, Juntendo University School of Medicine, Tokyo, Japan
7. Department of Pediatric Surgery, Graduate School of Medical Sciences, Kyushu University, Fukuoka, Japan
8. Department of Surgery, Division of Gastroenterological, General and Transplant Surgery, Jichi Medical University, Shimotsuke, Japan
9. Department of Pediatric Surgery, Nagoya University Graduate School of Medicine, Nagoya, Japan
10. Department of Pediatric Surgery, Tohoku University Graduate School of Medicine, Sendai, Japan
11. School of Pharmaceutical Sciences, Health Sciences University of Hokkaido, Hokkaido, Japan

*** Contact information for corresponding author:** Tatsuki Mizuochi, MD, PhD, Department of Pediatrics and Child Health, Kurume University School of Medicine, 67 Asahi-machi, Kurume, 8300011, Japan.

Tel.: +81-942 317565; fax: +81-942 381792; email: mizuochi_tatsuki@kurume-u.ac.jp

Running title: Oxysterols in biliary atresia

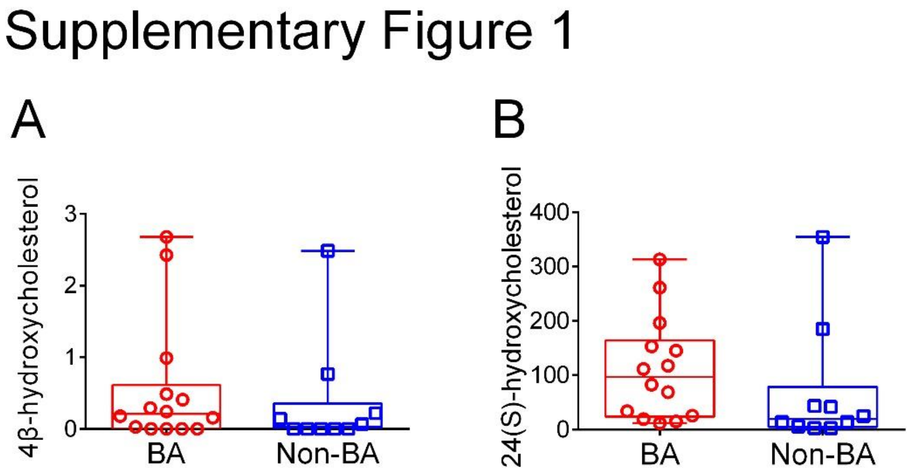

Supplementary Figure 1. Urinary 4β-hydroxycholesterol and 24(S)-hydroxycholesterol in BA and non-BA.

Urinary 4β-hydroxycholesterol [A] and 24(S)-hydroxycholesterol [B] are compared between biliary atresia (BA) and non-biliary atresia cholestatic controls (non-BA). Units are μmol/mol creatinine. Horizontal lines in the middle of boxes indicate medians, while tops and bottoms of boxes represent 75th and 25th percentiles, respectively. Whiskers above and below boxes represent maximum and minimum, respectively.

Supplementary Figure 2

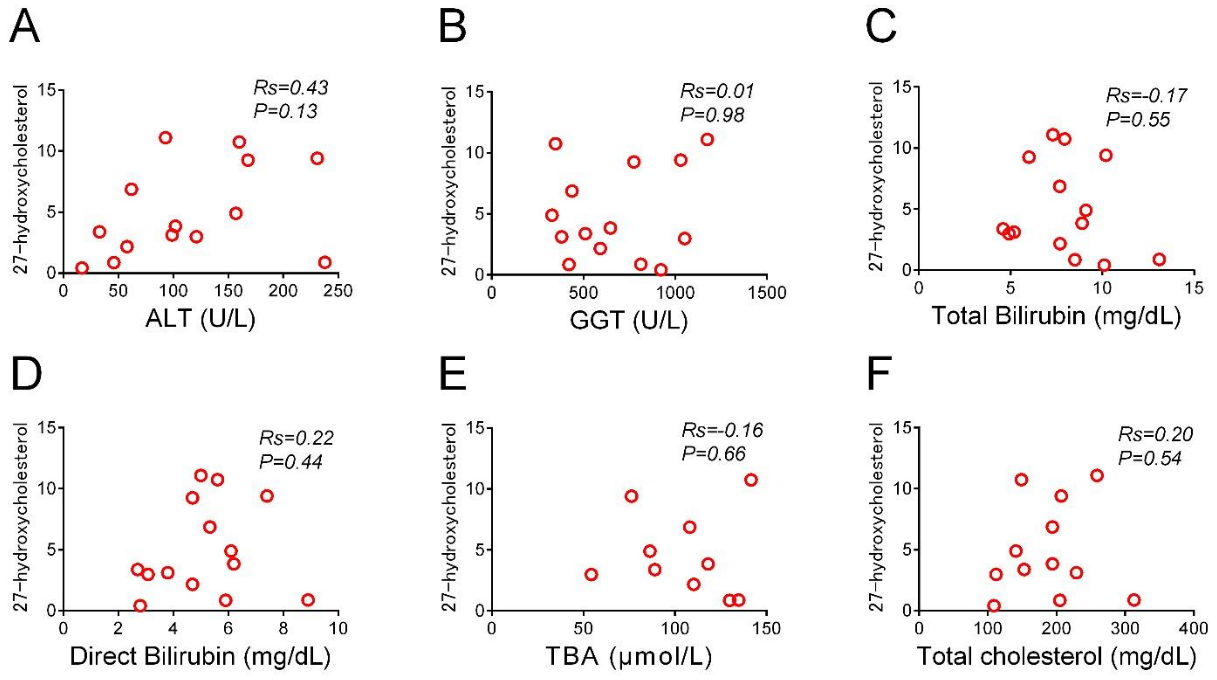

Supplementary Figure 2. Correlations between urinary 27-hydroxycholesterol and blood test results.

Correlations are shown between urinary 27-hydroxycholesterol and blood test results including serum alanine aminotransferase (ALT) [A], γ-glutamyltransferase (GGT) [B], total and direct bilirubin [C and D], total bile acids (TBA) [E], and total cholesterol [F] in patients with biliary atresia. *Rs*, Spearman's rank correlation coefficient.

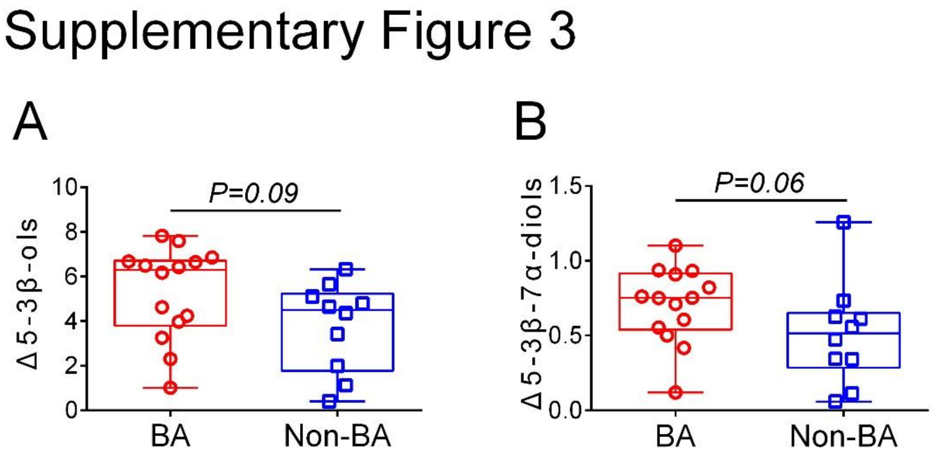

Supplementary Figure 3. Urinary Δ^{5}-3β-ols and Δ^{5}-3β,7α-diols compared between BA and non-BA.

Urinary 3β-hydroxy-5-cholenoic acids (Δ^{5}-3β-ols) [A] and 3β,7α-dihydroxy-5-cholenoic acids (Δ^{5}-3β,7α-diols) [B] are compared between biliary atresia (BA) and non-biliary atresia cholestatic controls (non-BA). Units are mmol/mol creatinine. Horizontal lines in the middle of boxes indicate medians, while tops and bottoms of boxes represent 75th and 25th percentiles, respectively. Whiskers above and below boxes represent maximum and minimum, respectively.

Supplementary Methods

Bile acid analysis by LC/ESI-MS/MS

We quantitatively analyzed 97 urinary bile acids by LC/ESI-MS/MS. To evaluate the acidic pathway, we analyzed bile acids such as 3β-hydroxy-5-cholenoic acid (Δ^5-3β-ol), glyco 3β-hydroxy-5-cholenoic acid (G-Δ^5-3β-ol), tauro 3β-hydroxy-5-cholenoic acid (T-Δ^5-3β-ol), 3β-hydroxy-5-cholenoic acid 3-sulfate (Δ^5-3β-ol-3S), glyco 3β-hydroxy-5-cholenoic acid 3-sulfate (G-Δ^5-3β-ol-3S), tauro 3β-hydroxy-5-cholenoic acid 3-sulfate (T-Δ^5-3β-ol-3S), 3β,7α-dihydroxy-5-cholenoic acid (Δ^5-3β,7α-diol), glyco 3β,7α-dihydroxy-5-cholenoic acid (G-Δ^5-3β,7α-diol), tauro 3β,7α-dihydroxy-5-cholenoic acid (T-Δ^5-3β,7α-diol), 3β,7α-dihydroxy-5-cholen-24-oic acid 3-sulfate (Δ^5-3β,7α-diol-3S), glyco 3β,7α-dihydroxy-5-cholenoic acid 3-sulfate (G-Δ^5-3β,7α-diol-3S), and tauro 3β, 7α-dihydroxy-5-cholenoic acid 3-sulfate (T-Δ^5-3β,7α-diol-3S), which were biosynthesized only in the acidic pathway. The Δ^5-3β-ols include Δ^5-3β-ol, G-Δ^5-3β-ol, T-Δ^5-3β-ol, Δ^5-3β-ol-3S, G-Δ^5-3β-ol-3S and T-Δ^5-3β-ol -3S. The Δ^5-3β,7α-diols include Δ^5-3β,7α-diol, G-Δ^5-3β,7α-diol, T-Δ^5-3β,7α-diol, Δ^5-3β,7α-diol-3S, G-Δ^5-3β,7α-diol-3S and T-Δ^5-3β,7α-diol-3S.

Bile acid sample preparation

Deuterium-labeled internal standard solutions of d4-CA, d4-GCA, d4-TCA d4-GCDCA, d4-TCDCA, d5-CDCA-3S, d5-GCDCA-3S, and d5-TCDCA-3S were mixed equally at a final concentration of 100 nmol/mL. A 10-uL volume of this mixture was added to 100 uL of the urine sample. The solution was transferred onto a solid-phase extraction cartridge (InertSep C18-B 100 mg/1 mL) that had been preconditioned with 1 mL of methanol and 3 mL of H_2O. After loading the sample, the column was washed with 1 mL of H_2O before the desired bile acids were eluted with 1 mL of 90% ethanol. After evaporation of the solvent, the residue was dissolved in 1 mL of 50% ethanol; 20 uL of the solution was injected into the LC/ESI-MS/MS. Urinary concentrations of individual bile acids were corrected for creatinine concentration and expressed as micromoles per moles of creatinine.
